# Community-Based *SERPINA1* Genotyping in an Isolated Alpine Town Reveals Heterozygous Pi*Mheerlen Carriers: Implications for Targeted Screening

**DOI:** 10.3390/biomedicines14071545

**Published:** 2026-07-10

**Authors:** Beatrice Ragnoli, Carlotta Bertelegni, Xheni Veselagu, Fausto Chiazza, Mario Malerba

**Affiliations:** 1 Respiratory Unit, S. Andrea Hospital, 13100 Vercelli, Italy; beatrice.ragnoli@aslvc.piemonte.it (B.R.); xheni.veselagu@aslvc.piemonte.it (X.V.); 2 Department of Translational Medicine, University of Piemonte Orientale, 28100 Novara, Italy; 3 Department of Pharmaceutical Sciences, University of Piemonte Orientale, 28100 Novara, Italy; 20037594@studenti.uniupo.it (C.B.); fausto.chiazza@uniupo.it (F.C.)

**Keywords:** alpha-1 antitrypsin deficiency, *SERPINA1*, genetic screening, rare variants, founder effect, population prevalence, Pi*Mheerlen variant, Northern Italy

## Abstract

**Background/Objectives:** Alpha-1 antitrypsin deficiency (AATD) is an underdiagnosed hereditary disorder that predisposes individuals to lung and liver disease. While its prevalence is higher in Northern Europe, data from specific, isolated populations in other regions are scarce. This study assessed the prevalence of pathogenic *SERPINA1* variants in the adult population of Ponte di Legno, a secluded town in the Italian Alps, to investigate potential founder effects involving rare genotypes. **Methods:** A cross-sectional community-based screening was conducted. Adult residents without previously diagnosed chronic respiratory diseases were invited to undergo spirometry and provide venous blood samples for serum AAT and CRP measurement. Buccal swabs were collected for genotyping, which was performed using a validated multiplex Luminex xMAP assay detecting 14 common and rare *SERPINA1* variants, with isoelectric focusing and Sanger sequencing for further characterization when required. **Results:** Ninety-one subjects were enrolled (median age 61 years; 37.4% male). Five individuals (5.5%) carried pathogenic *SERPINA1* variants: one Pi*MS heterozygote (1.1%), two Pi*MZ heterozygotes (2.2%), and two individuals heterozygous for the rare Pi*Mheerlen variant (Pi*M/Mheerlen) (2.2%). Median serum AAT levels were significantly lower in carriers of deficient alleles compared with Pi*MM individuals (100 mg/dL vs. 125 mg/dL, *p* = 0.0218). **Conclusions:** This community-based screening revealed a notable prevalence of AATD carriers in a geographically isolated Italian community, including two cases of the rare Pi*Mheerlen variant, suggesting a possible local founder effect. These findings underscore the value of targeted screening programs using rare variant panels to uncover hidden genetic architectures in isolated populations.

## 1. Introduction

Alpha-1 antitrypsin deficiency (AATD) is a hereditary autosomal co-dominant disorder caused by mutations in the *SERPINA1* gene, leading to reduced serum levels and/or dysfunction of the alpha-1 antitrypsin (AAT) protein [1]. AAT is a protease inhibitor primarily produced by the liver, and its principal function is to protect lung tissue from proteolytic degradation by neutrophil elastase [2]. Consequently, severe AATD predisposes individuals to early-onset pulmonary emphysema and bronchiectasis. In some cases, the accumulation of misfolded AAT polymers in hepatocytes can lead to liver disease, including cirrhosis and hepatocellular carcinoma (HCC) [3].

The normal *SERPINA1* allele is designated Pi*M. The most common deficiency alleles are Pi*S and Pi*Z, which, in various combinations (e.g., Pi*ZZ and Pi*SZ), account for the majority of clinically significant AATD cases [4]. However, over 150 other rare and null variants have been described, contributing to the complexity of the disease spectrum [5]. Despite being a treatable condition, AATD is frequently underdiagnosed, with affected individuals often misdiagnosed with chronic obstructive pulmonary disease (COPD) or asthma, leading to diagnostic delays of several years [6,7].

International guidelines recommend testing for AATD in all patients with COPD, unexplained bronchiectasis, or liver disease of unknown etiology [8]. Early diagnosis is crucial as it allows for the implementation of risk-reduction strategies (e.g., smoking cessation) and, in eligible patients with severe deficiency and established emphysema, the initiation of AAT augmentation therapy [9,10]. While evidence for augmentation therapy is strongest for Pi*ZZ individuals, its role in other genotypes like Pi*SZ is an area of active investigation [11].

Among the numerous rare *SERPINA1* variants, Pi*Mheerlen (p.Pro393Leu) is of particular interest because it causes complete intracellular retention of the AAT protein through a mechanism analogous to that of the Pi*Z allele, resulting in both loss of circulating antiprotease protection and potential hepatotoxicity from intracellular polymer accumulation [5]. However, its clinical significance in the heterozygous state remains poorly characterized due to its rarity.

The global prevalence of AATD varies, with the highest frequency of the Pi*Z allele observed in populations of Northern European descent [12]. In Italy, national and regional registries, coordinated within broader European frameworks like the European Alpha-1 Research Collaboration (EARCO) [13], have been established to map the disease [14]. However, epidemiological data from specific, geographically isolated alpine communities, where founder effects may alter local allele frequencies, remain limited [15,16].

This study was designed to investigate the prevalence of AATD-associated *SERPINA1* variants in a community-based sample of Ponte di Legno, a secluded town in the Lombardy region of the Italian Alps, to better understand the local genetic architecture and identify potential founder effects involving rare genotypes.

## 2. Materials and Methods

### 2.1. Study Design and Population

We conducted a cross-sectional, community-based screening study in Ponte di Legno, a town in the province of Brescia, Lombardy, Italy, with a resident population of approximately 1729 inhabitants. The screening activity conducted on 5–6 March 2022 was organized as a public health initiative (free open spirometry day). The use of anonymized data for research purposes was previously approved by the Institutional Review Board (Comitato Etico Interaziendale di Alessandria; protocol CE No. 6651, approved on 2 March 2022). All participants provided written informed consent for the use of their data for research purposes.

Participants were considered part of a generally healthy population based on the absence of previously diagnosed chronic respiratory diseases (COPD or asthma) and self-reported medical history collected through a structured questionnaire. The inclusion criteria were: age ≥18 years, residence in Ponte di Legno, participation in the screening program, and capacity to provide written informed consent. The exclusion criteria were: inability to perform acceptable spirometry according to ATS/ERS standards, acute respiratory infection at the time of screening, and refusal or inability to provide informed consent.

### 2.2. Data Collection and Procedures

Participants underwent baseline spirometry according to American Thoracic Society/European Respiratory Society (ATS/ERS) standards [17], using portable spirometers (Spirolab^®^, Medical International Research, MIR, Rome, Italy, version number MIR-911080) operated by trained personnel. Demographic information, smoking history, and known respiratory diagnoses were collected through a structured questionnaire. During the two screening days, buccal swabs were obtained from all participants, providing a non-invasive source of epithelial genomic DNA for *SERPINA1* analysis. Participants who provided written informed consent were subsequently invited to undergo venous blood collection through their primary care physician, exclusively for the measurement of serum AAT concentration and C-reactive protein (CRP), the latter quantified using a nephelometric method.

### 2.3. Laboratory Analysis

All genetic analyses were performed at the “Center for Diagnosis of Inherited Alpha-1 Antitrypsin Deficiency,” IRCCS Policlinico San Matteo Foundation, Pavia, Italy, following a validated diagnostic algorithm for the molecular characterization of *SERPINA1* variants [17]. DNA extraction from buccal swabs was successful in all 91 samples (100%). Genomic DNA extracted from buccal swabs was analyzed using the A1AT Genotyping Test (Progenika Biopharma, a Grifols company, Derio, Spain), a multiplex Luminex xMAP^®^ assay that simultaneously detects 14 common and rare pathogenic *SERPINA1* variants, including PiS, PiZ, and Pi*Mheerlen [18,19]. The genotyping call rate using the Luminex xMAP assay was 98%, with all samples yielding interpretable results after standard quality control procedures. Protein phenotyping was performed by isoelectric focusing (IEF) on an agarose gel to confirm genotyping results.

Sanger sequencing of the *SERPINA1* coding exons (II–V) was performed as a confirmatory step in cases of discordance between quantitative, phenotyping, and genotyping findings, or when a rare variant not included in the Luminex panel was suspected, in accordance with the diagnostic algorithm adopted at the Pavia reference center.

### 2.4. Statistical Analysis

Data were analyzed using GraphPad Prism (version 9.0, GraphPad Software, San Diego, CA, USA). Normality of continuous variables was assessed using the Shapiro–Wilk test. As most variables were not normally distributed (*p* ≤ 0.05), data are presented as median (Q1–Q3). Categorical variables are expressed as absolute values (*n*) and percentages (%). Comparisons between groups for continuous variables were performed using the Mann–Whitney U test; only AAT levels showed statistical significance. Categorical variables were analyzed using Fisher’s exact test. A *p*-value < 0.05 was considered statistically significant. The Mann–Whitney U test was selected for between-group comparisons because continuous variables did not follow a normal distribution (Shapiro–Wilk *p* ≤ 0.05). Fisher’s exact test was used for categorical variables given the small expected cell counts. Exact binomial 95% confidence intervals were calculated for proportions. Given the small sample size of the carrier group (*n* = 5), these analyses should be considered exploratory and hypothesis-generating rather than confirmatory.

## 3. Results

### 3.1. Cohort Characteristics

A total of 91 subjects consented to participate and were included in the analysis. The demographic and clinical characteristics of the study population are summarized in Table 1. The median age was 61 years (IQR 54–67), and 34 subjects (37.4%) were male. A majority of participants (67.0%) had a history of smoking. The population was mostly without previously diagnosed respiratory disease, with only two subjects (2.2%) reporting a known diagnosis of asthma and none reporting COPD. Lung function was within the normal range for the overall cohort, with a median FEV1 of 96% predicted (IQR 90–102) and a median FEV1/FVC ratio of 78% (IQR 74–84). C-reactive protein (CRP) values were also within the normal range, with a median of 0.2 mg/dL (IQR 0.2–0.4).

### 3.2. Genetic Screening Results

**Figure 1:**
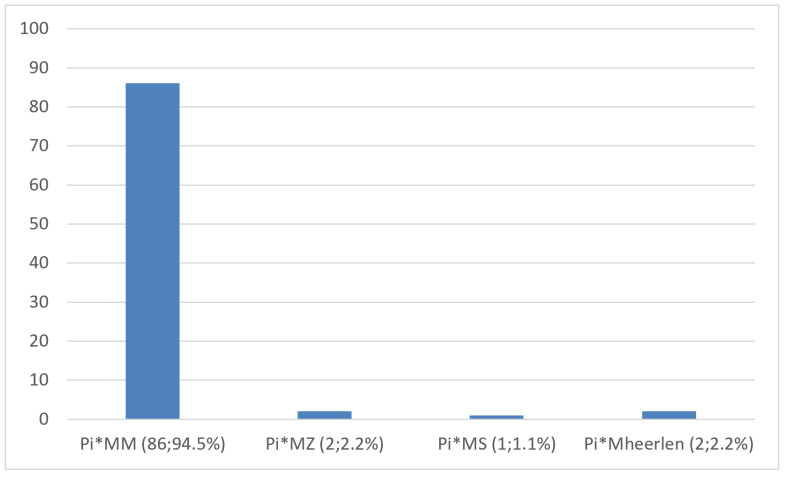
Distribution of *SERPINA1* genotypes in the Ponte di Legno study population (*n* = 91). The majority of individuals (94.5%) had the normal Pi*MM genotype. Pathogenic variants, including Pi*MS, Pi*MZ, and the rare heterozygous Pi*Mheerlen, were identified in 5.5% of the cohort.

Of the 91 participants, 86 (94.5%) were identified as having the normal Pi*MM genotype. The screening revealed 5 subjects (5.5%) carrying at least one pathogenic *SERPINA1* variant. The distribution of genotypes is illustrated in Figure 1. The identified deficiency genotypes comprised one Pi*MS heterozygote (1.1%), two Pi*MZ heterozygotes (2.2%), and, notably, two individuals heterozygous for the rare Pi*Mheerlen variant (Pi*M/Mheerlen; 2.2%).

The median serum AAT level was significantly lower in the group of subjects carrying a deficiency allele (*n* = 5) compared to those with the Pi*MM genotype (*n* = 86): 100 mg/dL (IQR 95–115) vs. 125 mg/dL (IQR 107–142), respectively (*p* = 0.0218). No statistically significant differences were observed between the two groups with respect to age, sex, smoking history, lung function parameters, or CRP levels (all *p* > 0.05), indicating that the two groups were clinically comparable in this small sample. The individual characteristics of the five subjects with pathogenic variants are detailed in Table 2. Given the small sample size, the 95% confidence interval for the observed 5.5% prevalence of pathogenic variant carriers (5/91) ranges from 1.8% to 12.4% (exact binomial method), indicating that the true population prevalence should be interpreted with caution.

## 4. Discussion

The most striking finding of our community-based screening in the secluded alpine town of Ponte di Legno is the identification of two individuals heterozygous for the rare Pi*Mheerlen allele. The Mheerlen variant (p.Pro393Leu) is a deficiency allele characterized by complete intracellular retention of the protein, similar to the Pi*Z variant, and is thus associated with a severe reduction in serum AAT levels and an increased risk for lung disease in the homozygous state [20]. Finding heterozygous individuals in a small, unselected cohort of 91 people is noteworthy and suggests a possible local founder effect. The AAT levels observed in these two subjects (90 and 115 mg/dL) are perfectly consistent with a heterozygous state, where the functional M allele provides partial AAT production. Both Pi*M/Mheerlen carriers were clinically well, with normal spirometry and no respiratory symptoms beyond occasional cough in one subject. While heterozygosity for the Mheerlen variant is not expected to cause severe disease in isolation, these individuals may be at increased risk if exposed to additional environmental insults (e.g., smoking, occupational exposures) or if they carry additional undetected modifying variants. Long-term clinical follow-up is warranted. This highlights the unique value of screening in geographically isolated populations to uncover rare genetic architectures that might be missed in larger, more heterogeneous national surveys.

Indeed, rare deficient *SERPINA1* variants represent a substantial proportion of cases in the Italian registry, accounting for 11% of index subjects with severe AATD and 13% of subjects with severe or intermediate AATD considered together. Importantly, the geographic clustering of rare *SERPINA1* variants in the Italian registry [21] suggests that isolated communities across the peninsula may each harbor distinct local variant enrichment. Furthermore, recent expanded carrier screening studies in Eastern Europe have also identified the Pi*Mheerlen variant. In western Romania, a population-based study found that *SERPINA1* falls among the genes with high carrier frequency (>1:50), with the Pi*Mheerlen variant (p.Pro393Leu) identified in 4 heterozygous individuals [22]. A subsequent study of reproductive couples from the same region identified the Pi*Mheerlen variant in the heterozygous state in both members of a couple, placing them at reproductive risk [23]. These findings confirm that this rare allele is present across geographically distant European populations and support the hypothesis of shared ancestral origins or independent recurrence events. Our findings provide preliminary community-level evidence for this phenomenon, demonstrating a pattern that can only be revealed through systematic screening using panels capable of detecting rare alleles. The identification of the same rare variant in geographically distant populations—an isolated alpine community in northern Italy and western Romania—raises intriguing questions about shared ancestral migration routes across Central and Southern Europe and warrants future collaborative studies integrating genomic data with historical demographic records to reconstruct the origins and dispersal patterns of rare *SERPINA1* alleles.

The diagnostic approach employed in this study is particularly noteworthy in this context. The use of the A1AT Genotyping Test (Progenika, Grifols), a Luminex xMAP-based multiplex assay validated at the Italian reference laboratory for AATD [18], allowed for the simultaneous detection of 14 *SERPINA1* variants. Crucially, the Pi*Mheerlen variant is included in this panel, enabling its identification without the need for additional sequencing. This workflow, which has been successfully deployed across a multinational diagnostic network [19], represents a robust tool for uncovering hidden genetic diversity in community screening programs.

Beyond the rare variant discovery, our screening revealed an overall 5.5% prevalence of individuals carrying pathogenic *SERPINA1* variants. This proportion is consistent with, and potentially slightly higher than, the range expected from published population surveys. De Serres and colleagues [24] calculated that Pi*S and Pi*Z allele frequencies in Italy translate into roughly 6–7% of Italians being heterozygous for Pi*MS or Pi*MZ, with northern Italian regions often showing Pi*S frequencies of about 3–4% and Pi*Z frequencies up to 1.5%. The enrichment observed in our isolated alpine population further supports the hypothesis that AATD can be more common in geographically secluded communities due to genetic drift [25].

Regardless of whether this represents a true excess above the national baseline, the identification of five previously undiagnosed carriers in a cohort of 91 volunteers underscores the persistent gap between estimated and diagnosed AATD prevalence—a gap that has been documented across European countries [7] and that this study makes tangible at the community level.

Our study reinforces the concept that AATD is not merely a ‘rare disease’ but a ‘rarely diagnosed’ one [6]. By screening a community-based sample, we identified five individuals who were previously unaware of their genetic risk. These individuals can now benefit from standard medical counseling regarding lifestyle modifications, particularly smoking cessation, avoidance of occupational exposures, and the need for regular clinical surveillance [26], with the aim of reducing risk and slowing disease progression.

This study has several limitations. First, the sample size is small and represents a fraction of the town’s total population, which may limit the precision of our prevalence estimates. Indeed, the 95% confidence interval for the observed carrier prevalence (1.8–12.4%) is wide, reflecting the inherent imprecision of estimates derived from small cohorts. Second, participation was voluntary, which may introduce selection bias. Individuals with pre-existing respiratory concerns may have been more motivated to attend, potentially inflating the observed carrier prevalence. Conversely, severely affected individuals may have been less likely to participate in a community event. However, the cohort demonstrated largely normal lung function and low CRP values, and no participant had a prior diagnosis of COPD, suggesting that the sample is reasonably representative of a healthy adult population rather than a high-risk group. Third, no validated symptom scores (e.g., CAT or mMRC) were systematically collected, which may limit the precise characterization of respiratory symptoms, and the definition of ‘healthy’ was based on the absence of previously diagnosed respiratory diseases. Fourth, the cross-sectional design does not allow for longitudinal follow-up to assess the clinical evolution of the identified carriers. In addition, although buccal swabs provide a practical and fully adequate source of genomic DNA for population screening, they yield lower DNA quantities compared with venous blood. This may reduce the efficiency of downstream analyses in a minority of samples, particularly when confirmatory sequencing is required. Nevertheless, all samples in this study provided sufficient DNA for complete genotyping. Finally, the absence of family history data precludes an analysis of the genealogical origins of the Mheerlen variant in this community. Future studies combining genealogical records with extended family screening could determine whether the Pi*Mheerlen heterozygosity observed here reflects a common founder ancestor, which would have significant implications for targeted cascade testing in the region.

Despite these limitations, the strengths of our study include its community-based approach, which avoids the ascertainment bias inherent in studies restricted to high-risk groups such as COPD patients, and the use of a comprehensive, validated molecular diagnostic workflow capable of identifying both common and rare variants.

## 5. Conclusions

In conclusion, our community-based screening program in a secluded northern Italian alpine town identified a 5.5% prevalence of pathogenic *SERPINA1* variant carriers, all of whom were previously undiagnosed. Most strikingly, two individuals were found to be heterozygous for the rare Pi*Mheerlen variant (Pi*M/Mheerlen), a finding suggesting a possible local founder effect and one that would not have been captured by standard clinical screening restricted to symptomatic or high-risk patients. These results demonstrate that population-based AATD screening, using comprehensive genotyping panels that include rare variants, is feasible and potentially informative. They further suggest that geographically isolated communities in Italy may represent a target worth exploring. Genealogical studies and cascade family testing in this population are warranted to clarify the origin and clinical significance of the Pi*Mheerlen variant and to inform regional screening strategies.

## Figures and Tables

**Table 1:** Demographic and clinical characteristics of the study population.

Variable	All Subjects (*n* = 91)
Age, years	61 (54–67)
Male sex, *n* (%)	34 (37.4)
Smoking history, *n* (%)	61 (67.0)
FEV1, % predicted	96 (90–102)
FEV1/FVC, %	78 (74–84)
AAT, mg/dL	120 (104–140)
CRP, mg/dL	0.2 (0.2–0.4)
Asthma, *n* (%)	2 (2.2)
COPD, *n* (%)	0 (0)

Data are presented as follows: continuous variables are expressed as the median (first–third quartiles) (IQR), and categorical variables are expressed as *n* (%). Abbreviations: FEV1%, forced expiratory volume in 1 s; FVC, forced vital capacity; AAT, alpha-1 antitrypsin; CRP, C-reactive protein; COPD, chronic obstructive pulmonary disease.

**Table 2:** Characteristics of subjects with pathogenic *SERPINA1* variants (*n* = 5).

Genotype Pi	(Patient 1) Pi*MS	(Patient 2) Pi*MZ	(Patient 3) Pi*MZ	(Patient 4) Pi*MHeerlen	(Patient 5) Pi*MHeerlen
AAT (mg/dL)	100	95	119	90	115
Age, years	56	56	79	76	54
Sex	F	F	M	M	F
Smoking habit	No	Yes	Yes	Yes	Yes
FEV1%	106	90	80	90	93
FEV1/FVC%	84	72	79	76	84
CRP mg/dL	0.4	0.5	0.1	0.2	0.5
Asthma or COPD	No	No	No	No	No
Symptoms					
Chronic cough with/without sputum	No	Yes (cough)	Yes (cough)	Yes (cough + sputum)	Yes (cough + sputum)
Non-respiratory comorbidities	Hypothyroidism	Hypertension	Hypertension Carotid atheromasia	Hypertension Dyslipidemia	No
Family history of respiratory and liver diseases	No	Yes (COPD)	Not known	Yes (pulmonary emphysema)	Not known

Abbreviations: FEV1%, forced expiratory volume in 1 s; FVC, forced vital capacity; AAT, alpha-1 antitrypsin; CRP, C-reactive protein. COPD, chronic obstructive pulmonary disease.

## Data Availability

The data presented in this study are available upon request from the corresponding author. The data are not publicly available due to privacy and ethical restrictions.

## Abbreviations

The following abbreviations are used in this manuscript:

AAT	Alpha-1 antitrypsin
AATD	Alpha-1 antitrypsin deficiency
ATS	American Thoracic Society
COPD	Chronic obstructive pulmonary disease
CRP	C-reactive protein
DNA	Deoxyribonucleic acid
EARCO	European Alpha-1 Research Collaboration
ERS	European Respiratory Society
FEV1	Forced expiratory volume in 1 s
FVC	Forced vital capacity
HCC	Hepatocellular carcinoma
IEF	Isoelectric focusing
IQR	Interquartile range (first–third quartile)
IRCCS	Scientific Institute for Research, Hospitalization and Healthcare
Pi	Protease inhibitor
PiMM	Normal AAT phenotype (M/M alleles)
PiMS	Heterozygous AAT phenotype (M/S alleles)
PiZZ	Severe AAT phenotype (Z/Z alleles)
